# Modeling DNA Methylation Profiles and Epigenetic Analysis of Safflower (*Carthamus tinctorius* L.) Seedlings Exposed to Copper Heavy Metal

**DOI:** 10.3390/toxics11030255

**Published:** 2023-03-09

**Authors:** Ekrem Bölükbaşı, Mehmet Karakaş

**Affiliations:** 1 Department of Environmental Protection and Technologies, Suluova Vocational School, Amasya University, Amasya 05100, Türkiye; 2 Central Research Laboratory, Amasya University, Amasya 05100, Türkiye; 3 Department of Biology, Faculty of Science, Ankara University, Ankara 06100, Türkiye

**Keywords:** *Carthamus tinctorius*, heavy metal, copper toxicity, epigenetic, ecotoxicology

## Abstract

Heavy metals are chemical elements with high density that can be toxic or poisonous even at low concentrations. They are widely distributed in the environment due to industrial activities, mining, pesticide use, automotive emissions and domestic wastes. This study aimed to investigate the toxic effects of copper (Cu) heavy metal on safflower plants in terms of genetic and epigenetic parameters. Safflower seeds were exposed to different concentrations of Cu heavy metal solution (20, 40, 80, 160, 320, 640, 1280 mg L^−1^) for three weeks, and changes in the genomic template stability (GTS) and methylation pattern in the root tissues were analyzed using PCR and coupled restriction enzyme digestion-random amplification (CRED-RA) techniques. The results indicated that high doses of Cu have genotoxic effects on the genome of safflower plants. Epigenetic analysis revealed four different methylation patterns, with the highest total methylation rate of 95.40% observed at a 20 mg L^−1^ concentration, and the lowest rate of 92.30% observed at 160 mg L^−1^. Additionally, the maximum percentage of non-methylation was detected at 80 mg L^−1^. These results suggest that changes in the methylation patterns can serve as an important mechanism of protection against Cu toxicity. Furthermore, safflower can be used as a biomarker to determine the pollution in soils contaminated with Cu heavy metal.

## 1. Introduction

**Figure 1:**
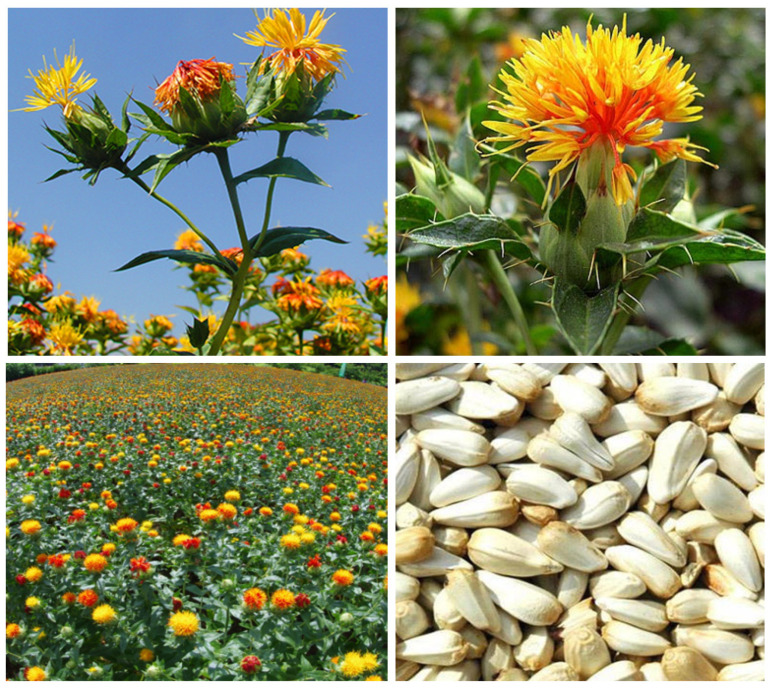
A comprehensive perspective; safflower plants, its flowers, fields and seeds [5].

Safflower (*Carthamus tinctorius* L.) is a plant species from the Asteraceae family, widely cultivated for its economic significance as an oil plant. There are numerous cultivated varieties of safflower found worldwide, consisting of approximately 25 different species. Safflower is an annual herb with broad leaves and produces flowers in various colors, including yellow, red, orange, white and cream [1,2]. The seeds of safflower are a valuable source of high-quality oil, typically containing between 30–50%. Studies have demonstrated that safflower oil has superior quality compared to other oil crops, such as soybean, sunflower and corn. One of the distinguishing features of safflower oil is its high content of linoleic acid, which is approximately 70% in comparison to other oilseed products [3,4] (Figure 1).

In order to survive, all living things are constantly in contact with the physical elements of the ecosystem. They may encounter negative situations against any changes that may occur in their environment. While humans and animals can react more quickly to adversity or are easier to move away from, this is the opposite of plants. It is completely vulnerable to the negative effects that may occur around the plants. As the most important natural food sources of people in the food chain are plants, people can also suffer indirectly from these problems [6].

Commercial oil crops, such as safflower, soybean, rapeseed, sunflower, poppy, peanut and sesame, are often exposed to various abiotic stresses such as drought, low temperature, salinity, excessive water and heavy metal contamination [7]. Among these stresses, heavy metal pollution is a significant global environmental and health problem affecting a broad range of organisms, including microorganisms, plants, animals and humans. Heavy metals can accumulate intensely in soil and water, posing a common environmental concern that requires urgent action [8,9,10]. The sources of heavy metal pollution are numerous, including industrial activities, urban waste and the combustion of fossil fuels, spraying and fertilization in agriculture, the use of heavy metal-containing pesticides, mining and many other factors [11,12,13,14].

Heavy metal contamination in soil or water ecosystems can cause harm to the morphological, cytological, metabolic and genomic integrity of many organisms, including plants [15,16]. Certain heavy metals can enter plant cells through specific carriers and lead to the formation of reactive oxygen species in organelles, disrupting the metabolism through various redox reactions [17,18]. Although copper (Cu), zinc (Zn), manganese (Mn) and nickel (Ni) are required for normal plant growth and development at low concentrations [19,20], the excessive accumulation of these metals in plant tissues can cause significant damage to their morphology, cytology, metabolism and genomic integrity, ultimately affecting the quality of life of the plants [21,22].

Cu is known to be an essential micronutrient for plant nutrition [23], but an excess of Cu ions can have toxic effects and cause damage to biological molecules such as proteins, enzymes and nucleic acids in plants [24]. Additionally, Cu at high concentrations can damage most functional biomolecules, including membrane lipids, and lead to oxidative stress by inducing an increase in reactive oxygen species. Under such circumstances, various chemical, biological and physical agents can cause damage to genetic materials, such as DNA and RNA [25].

Epigenetics is a fascinating field of molecular biology that delves into the study of changes in gene expression that are not caused by alterations in DNA sequences. Despite the lack of changes in the DNA sequence, these modifications can still be inherited and passed down from generation to generation. This area of research focuses on investigating the inherited phenotypic variations that arise as a result of non-genetic environmental effects. These modifications can have a direct impact on the cell or organism without any alteration in the DNA sequence itself [26]. Overall, the study of epigenetics sheds light on how external factors can influence gene expression and how these effects can be passed down through generations.

DNA methylation is an essential enzymatic modification that involves the addition of a methyl group to the carbon-5 cytosine, and plays a crucial role in the epigenetic control mechanism of genes in plants. It is a heritable modification that can be passed down from generation to generation, and is also a reversible process that can return to the original structure. This makes DNA methylation an important mechanism that supports plant defenses [27,28,29]. The levels of methylcytosine in the genome can be detected using various techniques. The CRED-RA technique is a significant method used to study the methylation status in plants. The CRED-RA technique has been successfully applied in many studies involving different plant species to determine their methylation patterns [30,31,32,33,34,35,36].

The aim of this study was to investigate the potential genotoxicity of Cu heavy metal on safflower (*C. tinctorius* L.) seedlings, and to assess the possible methylation differences between the treatment groups and control plants using CRED-RA analysis, which is a PCR-based molecular indicator. Cu heavy metal is known to have adverse effects on plant growth and development and can cause damage to genetic materials such as DNA. Therefore, understanding the potential genotoxicity of Cu heavy metal on plants is of great importance. The results of this study could contribute to the development of strategies to mitigate the negative effects of heavy metal pollution on plant growth and development.

## 2. Materials and Methods

### 2.1. Hydroponic Growth of Safflower Seedlings and Cu Stress Treatments

All stages of this study were carried out at the Central Research Laboratory of Amasya University, Amasya, Turkey. The safflower seeds used for the experiment and their surfaces were sterilized using a 70% ethyl alcohol and 30% sodium hypochlorite solution before being planted in seedling trays filled with sterile perlite. The trays were divided into eight groups: one control and seven experimental (20, 40, 80, 160, 320, 640, 1280 mg L^−1^) groups, with different concentrations of Cu solution (CuSO_4_·5H_2_O, product nu: 209,198, Sigma-Aldrich Chemie GmbH, Taufkirchen, Germany). The seeds were planted in equal numbers in a seedling tray filled with sterile perlite. The safflower seedlings were grown hydroponically in pots containing 0.2 L of modified 1/10 Hoagland’s solution, which provided the necessary macronutrients, micronutrients and ions for growth; (macronutrients; 2 M KNO_3_ (202 g L^−1^), 2 M Ca(NO_3_)_2_·4H_2_O (472 g L^−1^), 2 M MgSO_4_·7H_2_O (493 g L^−1^), 1 M KH_2_PO_4_ (115 g L^−1^) micronutrients; H_3_BO_3_ (2.86 g L^−1^), MnCl_2_·4H_2_O (1.81 g L^−1^), ZnSO_4_·7H_2_O (0.22 g L^−1^), CuSO_4_·5H_2_O (0.08 g L^−1^), H_2_MoO_4_·H_2_O (0.02 g L^−1^), C_12_H_12_Fe_2_O_18_ (5 g L^−1^)) [37].

The safflower seedlings were incubated in a controlled environmental growth chamber in light with 250 mmol m^−2^s^−1^ photosynthetic photon flux at 25 °C and 70% relative humidity. All safflower cultivars were grown in the climatic chamber for 21 days. Within a 24 h period, 16 h (25 °C, 70% humidity) day and 8 h (22 °C, 60% humidity) night cycles were applied. The control group was treated only with 15 mL Hoagland’s solution, while the other groups were treated with increasing concentrations of the Cu solution (15 mL for each concentration). The irrigation process was carried out daily for the first three days, and then with a day break in between for the following days. All treatments lasted for 21 days; after harvesting, the root tissues of the safflower seedlings were immediately frozen in liquid nitrogen and stored at −20 °C until DNA extraction.

### 2.2. DNA Isolation and PCR Amplification Protocols

The roots of the safflower seedlings, weighing approximately ~200 mg each, were ground to a fine powder using liquid nitrogen. The total genomic DNA was extracted from the safflower samples using the DNeasy Plant Mini Kit (Cat. No: 69,104; Qiagen, Germany) according to the manufacturer’s protocol. The extracted DNA was quantified and the quality was evaluated using a Nanodrop Spectrophotometer (NanoDrop ND-1000 Spectrophotometer, Thermo Fisher Scientific, Waltham, MA, USA) and was also confirmed by gel electrophoresis with a 1.2% agarose containing 0.05 μL ml^−1^ ethidium bromide (0.5 μg ml^−1^). For the RAPD-PCR (Random Amplification of Polymorphic DNA) reaction, the DNA samples with suitable purity and concentration were selected. A standard PCR reaction mixture of 25 μL was prepared for each sample, which included 200 ng genomic DNA, 1× reaction buffer, 2.5 mmol MgCl_2_, 20 μmol dNTPs, 0.2 μmol primer, and 0.5 U Taq DNA polymerase (Promega, Germany). Ten random amplified polymorphic DNA (RAPD) primers (Operon Technologies Inc., Alameda, CA, USA) were used for the PCR reaction. The PCR reaction was performed under optimized conditions, which involved an initial denaturation step at 95 °C for 5 min, followed by 35 cycles of denaturation at 94 °C for 90 s, annealing at 36 °C for 60 s, and extension at 72 °C for 120 s, followed by a final extension at 72 °C for 5 min. A negative control was run with each sample to detect any contamination without the presence of a DNA template.

### 2.3. CRED-RA Assay

The methylation patterns of the genomic DNA in the control and treated groups were analyzed using CRED-RA with *MspI* (Cat. nu: ER0541, Thermo Fisher Scientific, Bremen, Germany) and *HpaII* (Cat. nu: ER0511, Thermo Fisher Scientific, Bremen, Germany) enzymes. The standard reaction volume for each sample was 20 μL, consisting of approximately 1 μg genomic DNA, 2 μL of 10× enzyme reaction buffer and 10 U mL^−1^ of restriction enzyme. After incubating the samples in an incubator at 37 °C for 4 h, the restriction enzymes were inactivated by holding the samples at 95 °C for 15 min. For the PCR reaction, approximately 200 ng of digestion product, 2.5 µL of 10× reaction preservative, 20 μmol dNTPs, 2.5 µL of MgCl_2_, 0.2 μmol primer and 0.7 U Taq polymerase (Promega, Walldorf, Germany) were used in a 25 µL reaction volume. Six out of ten RAPD primers showed monomorphic band profiles in the RAPD-PCR and were used for the CRED-RA assay. The optimized reactions included an initial denaturation step of 96 °C for 90 s, followed by 45 cycles of denaturation at 95 °C for 30 s, annealing at 36 °C for 60 s, extension at 72 °C for 120 s and a final extension period of 72 °C for 10 min.

### 2.4. Electrophoresis

All of the experiments were performed in triplicate and repeated thrice. The PCR products were separated with a 1.6% agarose gel containing 0.05 μL mL^−1^ ethidium bromide (0.5 μg mL^−1^) at 100 V for 90 min. A negative control was used for each group to test for contamination, and visualization was performed. To estimate the molecular weight of the fragments, a 100–1000 bp DNA ladder (Sigma Aldrich, P1473-1VL) was used. The gels were displayed with an UV imaging system (Gene Genius, Syngene, Sparta, TN, USA) and photographed using the GyneSnap Software (Synoptics Co., Cambridge, UK).

### 2.5. Statistical Analysis

The RAPD and CRED-RA banding patterns were analyzed using the TotalLab TL120 software (Nonlinear Dynamics Ltd. Newcastle, UK). Polymorphism in the RAPD profiles was expressed as the disappearance of a normal band and the appearance of a new band relative to the control. The average polymorphism was calculated for each experimental group (Cu treatment), and changes in these values were calculated as the percentage of their value in the control (set to 100%) [38].

After analyzing the RAPD profiles, we calculated the GTS (%) using the following formula: (1−a/n) × 100, where ‘a’ represents the number of polymorphic bands in each sample treated with different Cu solutions and ‘n’ represents the total number of bands in the control sample. Polymorphism was identified as the appearance or disappearance of bands in the treated sample’s RAPD profiles compared to the control RAPD profiles. Additionally, the polymorphism (%) was calculated as: (a/n) × 100, where ‘‘a’’ indicates number of polymorphic bands in the Cu treated sample and ‘‘n’’ is the number of total bands in the control sample.

### 2.6. Detection of Methylation Patterns by CRED-RA Analysis

To evaluate the data obtained from the CRED-RA analysis, Table 1 was used as a reference. The *HpaII* and *MspI* restriction enzymes used in the CRED-RA analysis have different abilities to digest DNA based on the methylation pattern of the cytosine. To analyze the results obtained from the CRED-RA, the amplified bands were categorized into four methylation types based on the presence or absence of digestions. This categorization was established by previous studies conducted by Liu et al. [31], Wang et al. [32], Cai et al. [36], Pan et al. [39] and Karan et al. [40]. For each methylation type, the methylation band profile was scored as either present (1) or absent (0), based on the amplification of the bands on the agarose gel. This scoring system enabled the determination of the DNA sample’s methylation pattern.

## 3. Results

### 3.1. Data Analysis of RAPD-PCR Results

In this study, the impact of Cu stress on safflower plants was investigated using RAPD-PCR analysis. The results revealed a significant degree of polymorphism among the safflower samples exposed to Cu stress. Out of the 15 RAPD primers used, ten of them revealed polymorphic bands that were distinct from those of the control group of safflower plants. Among these primers, *OPC* 09 (59.7%), *OPC* 07 (56.8%), *OPC* 11 (55.9%) and *OPC* 10 (54.1%) exhibited significant polymorphic band patterns (as shown in Table 2). These results demonstrate that these primers are effective indicators of the mutagenic effects of heavy metal Cu on safflower plants.

Based on the analysis of the GTS rates using the RAPD profiles, the results revealed that the highest rate was recorded at a 40 mg L^−1^ Cu concentration, with a value of 88.90%. In contrast, the lowest rate was observed at 320 mg L^−1^ Cu stress, with a GTS rate of 82.25% (as shown in Table 3). These findings provide clear evidence of the significance of varying Cu concentrations in inducing toxicity. The results suggest that increasing the Cu concentration may adversely affect the genetic makeup of the organism and reduce its ability to withstand the stress, as evidenced by the decreasing GTS rates.

### 3.2. CRED-RA Analysis

**Figure 2:**
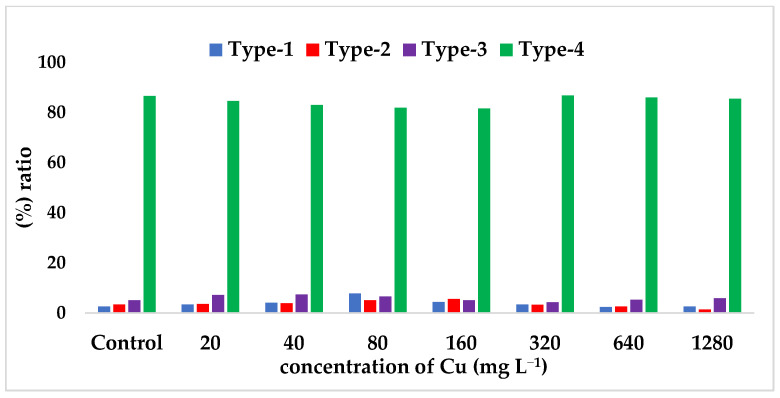
The average rates of methylation types based on CRED-RA data analysis in the root tissues of safflower seedlings after exposure to increasing Cu concentrations (20–1280 mg L^−1^ CuSO_4_·5H_2_O) for 21 days, compared to the control group (containing 0 mg L^−1^ Cu and irrigated only with Hoagland solution).

The results showed that Cu has a noteworthy impact on the epigenetic mechanisms, specifically in the form of DNA methylation differences in safflower plants. This plays a fundamental role in granting resistance to Cu stress in safflower plants. To detect the differences in the four different methylation types identified in safflower seedlings that were exposed to different concentrations of Cu, six different primers were analyzed individually. The PCR band profiles were evaluated against Table 1 to determine the methylation patterns and their differences. The methylation band profiles were then scored as yes/presence (1) or no/absence (0) according to Table 1. The scores of the different methylation types are presented in Table 4 and illustrated comparatively in Figure 2.

In this research study, we utilized the PCR-based CRED-RA technique to investigate the DNA methylation patterns in safflower seedlings exposed to varying concentrations of Cu stress. Our analysis revealed some interesting findings. Firstly, we observed that the Type-I methylation pattern, which represents non-methylated regions, had the highest rate of 7.80 at 80 mg L^−1^. This suggests that there are unmethylated regions in the safflower genome under all Cu stress concentrations, especially at 80 mg L^−1^. We also calculated the percent (%) ratio of the total methylation, full-methylation and semi-methylation by using all methylation types. The highest rate of the Type-II methylation pattern, which represents an externally methylated semi-methylation pattern of cytosine nucleotide on the DNA single strand, was detected at 5.60% at 160 mg L^−1^. Furthermore, we identified the Type-III and Type-IV methylation patterns, which represent the inner and outer methylated cytosine in both the helixes of DNA and the full methylation pattern, respectively. The highest rate of Type-IV methylation pattern was detected at 91.80 at 20 mg L^−1^, and this pattern was at the highest level in all different concentrations between 20 to 1280 mg L^−1^ compared to the other methylation patterns.

Figure 2 presents the (%) ratio of the different methylation types obtained from the CRED-RA analysis in safflower samples, allowing for a clear comparison of the changes in the methylation types based on the concentration variations. The results of this analysis reveal significant differences in the levels of methylation pattern between different concentrations of Cu stress, with specific methylation types exhibiting a higher percentage at certain concentrations. By examining the methylation ratios, it becomes possible to understand the impact of Cu stress on the epigenetic mechanisms of safflower plants. The comparative analysis presented in Figure 2 highlights the importance of Type-IV methylation, which had the highest value at all Cu concentrations, suggesting its potential role in providing resistance to Cu stress.

**Figure 3:**
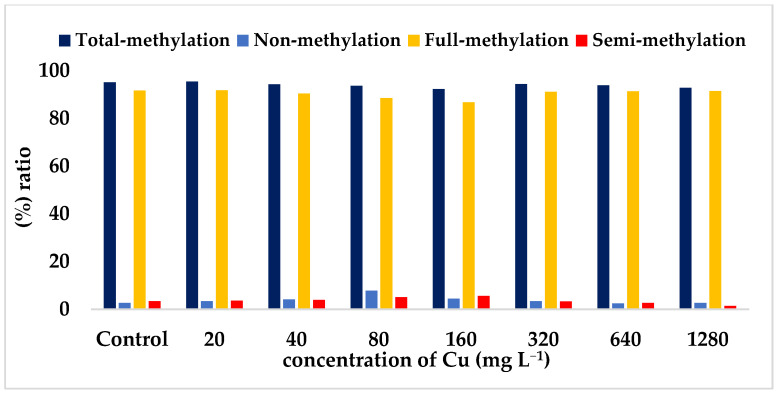
The average rates of methylation patterns based on CRED-RA data analysis in the root tissues of safflower seedlings after exposure to increasing Cu concentrations (20–1280 mg L^−1^ CuSO_4_·5H_2_O) for 21 days, compared to the control group (containing 0 mg L^−1^ Cu and irrigated only with Hoagland solution).

To facilitate a more comprehensive comparison of the differences between models, Figure 3 displays the (%) ratio of the various methylation pattern obtained from CRED-RA analysis in safflower samples. This comparative analysis provides a clear visual representation of the distinct differences in methylation patterns between different models. By analyzing the ratios of methylation pattern, it is possible to gain deeper insights into the mechanisms underlying plant responses to different concentrations of Cu stress.

## 4. Discussion

Heavy metal pollution is a prominent topic in the field of environmental pollution research. In high concentrations, heavy metals can have a devastating impact on a wide range of living organisms, making it a critical issue that needs to be addressed. One of the most significant consequences of heavy metal contamination is its ability to inhibit plant growth [22,23,24,25]. The excessive accumulation of heavy metals in plant tissues leads to negative impacts on seed germination and root and stem growth. Several studies have established the toxicity caused by heavy metals in high concentrations, acting as catalysts in the oxidative degradation of biological macromolecules, resulting in DNA damage through oxidative stress [31,33,41,42].

Safflower cultivation is highly dependent on effective weed control in the field prior to planting. During the initial 3–4 weeks of growth, safflower has relatively low competition with weeds, making it highly sensitive to any weed presence in the field. Therefore, it is recommended to use highly effective herbicides, such as trifluralin, metolachlor, EPTC, barban, profluralin and paraquat, to control weeds in the soil prior to planting safflower. However, it is important to note that many of these herbicides contain chemicals, such as copper ethylenediamine sulfate salts, copper triethanolamine complex, copper hydrazinium sulfate, copper sulfate, leadarsenite, copper arsenite, etc., which can have adverse effects on living organisms [43]. Therefore, in this study, the effects of Cu heavy metal have been investigated.

Cu is an important micronutrient for plant growth and development. However, excessive Cu can result in harmful effects on living organisms, particularly due to the production of reactive oxygen species (ROS) in cells. These free radicals can cause significant damage to various cellular components, including DNA. The damages to DNA can be extensive and varied, ranging between base deletions, pyrimidine dimers, cross-links, strand breaks and base modifications, such as methylation. Several studies, such as those by Tuteja et al. [44] and Nagajyoti et al. [45], have highlighted the potential DNA damage caused by Cu exposure.

Various molecular parameters or techniques that can be utilized to detect DNA degradation are also instrumental in identifying the genotoxic effects of heavy metals on plants. DNA fingerprinting techniques such as RAPD-PCR are widely used to identify DNA changes in plants due to contaminants such as heavy metals [38,39,40,41,42].

One of the main advantages of using the RAPD technique is its ability to detect various types of genetic alterations and damage, including point mutations, chromosomal rearrangements and deletions or insertions in the genomic DNA. Studies by Liu et al. [46] and Taspinar et al. [47] have shown that the RAPD technique is particularly useful for detecting such genetic changes. Additionally, it has been noted in studies by Atienzar et al. [48] and Bolukbasi and Aras [25] that the loss of normal PCR products can be indicative of genotoxin-induced DNA damage, point mutations or complex chromosomal rearrangements. Conversely, the appearance of new PCR products may suggest mutations in the oligonucleotide priming sites, changes due to large deletions or homologous recombination, as highlighted in studies [38,39,40,41,42]. In this study, the RAPD-PCR technique was employed to investigate the changes in the DNA band profiles in safflower plants exposed to varying concentrations of Cu heavy metal stress for a period of 21 days. Upon analysis, significant alterations were observed in the DNA band profiles of the experimental groups when compared to the control group. The RAPD-PCR data indicated the presence of a substantial amount of polymorphism in the samples subjected to Cu heavy metal stress. This difference in the RAPD profile is attributed to mutations occurring at the sites where the primers were bonded to the DNA structure, as reported in previous studies [38,39,40,41,42,45,46,47].

The findings of this study suggest that the primers used in RAPD-PCR are a powerful marker for detecting the mutagenic effects of Cu heavy metal in safflower plants, especially at high concentrations. Furthermore, the changes observed in the GTS rates highlight the significance of varying Cu stress concentrations. Additionally, it could also help in devising effective strategies for mitigating the impact of pollution on plants [45,46,47,49,50,51]. The results obtained from this study support the previous research on Cu toxicity conducted on plants such as hydrilla [52], seagrass [53] and moss *Scopelophila cataractae* [54].

Epigenetic mechanisms, such as DNA methylation, are crucial defense mechanisms utilized by plants to combat various abiotic stresses, including salinity, drought and heavy metal contamination. DNA methylation is a chemical modification that alters the DNA structure and is essential for regulating gene expression and genome defense in plants. This modification involves the enzymatic attachment of a methyl group to the fifth carbon of cytosine, catalyzed by DNA Methyltransferase enzymes, which play a vital role in controlling gene expression in plants [29,30,31,32,33,34,35,36,49].

Several studies have shown that stress conditions, such as heavy metal stress, can cause changes in the DNA methylation patterns [41,42,46]. For example, Choi and Sano found that aluminum stress led to changes in the DNA methylation levels in tobacco plants [55]. On the other hand, Erturk et al. determined the effect of Cu heavy metal on methylation differences in maize [56]. The methylation status has been reported to change in response to Cu stress in rice [57]. Similarly, significant changes in the methylation levels were observed in white spruce [58], lettuce [59] and *Silene paradoxa* [60] plants exposed to high concentrations of Cu heavy metal. In addition, Cong et al. investigated the effect of Cu on the methylation status of rice and found that rice plants treated with two different concentrations of Cu exhibited dose-dependent responses. The higher doses of Cu resulted in more hypomethylations at specific loci [61].

Interestingly, we found that safflower seedlings exposed to Cu stress were able to cope with the stress by altering their DNA methylation patterns. The total methylation pattern, consisting of Type-II, Type-III and Type-IV methylation, was observed at all Cu stress concentrations, with the highest level of 95.40 at 20 mg L^−1^. Notably, the analysis revealed that Type-IV methylation had the highest value at all Cu concentrations, indicating its importance in providing resistance to Cu stress in safflower plants. The present analysis, which determined the methylation status of safflower using the CRED-RA technique, reveals that increasing the concentrations of Cu is effective in inducing non-methylation and semi-methylation patterns, while inhibiting using the CRED-RA technique total metylation and full-methylation of the genome.

In summary, our research has uncovered fresh perspectives and new knowledge regarding the molecular mechanisms that govern safflower seedlings’ reaction to Cu-induced stress. The results of this study highlight the crucial role of different methylation types on the mechanism of the resistance to Cu stress in safflower plants and suggest that different methylation patterns may play different roles in this process.

## 5. Conclusions

The CRED-RA technique was used to analyze the DNA methylation patterns in safflower plants exposed to various concentrations of Cu heavy metal stress. The findings of the study showed significant changes in the methylation patterns and polymorphisms, suggesting that the plant’s defense mechanism is impacted by such stress. Interestingly, the degree of alteration in methylation was found to be a critical factor in determining the plant’s biodefense mechanism. Our research also revealed that, in elevated concentrations, Cu can be genotoxic for safflower plants and that the use of Cu-based herbicides in safflower cultivation should be approached with caution. It is our hope that this study will spur future research in the area of environmental pollution, contributing to a better understanding of the impacts of heavy metals on plant life.

## Figures and Tables

**Table 1:** Methylation types and patterns identified by *HpaII* and *MspI* restriction enzymes based on their digestion abilities.

Type	Methylation Status		*HpaII*	*MspI*	Score of Band Profile			
					x	y	z	
**Type I**	CCGG GGCC		digestion	digestion	−/1	+/0	+/0	Non-methylation
**Type II**	**CC**GG GGCC	**C**CGG GGCC	digestion	undigestion	−/1	+/0	−/1	Semi-methylation
**Type III**	C**C**GG GG**C**C		undigestion	digestion	−/1	−/1	+/0	Full-methylation
**Type IV**	**CC**GG GG**CC**		undigestion	undigestion	−/1	−/1	−/1	Full-methylation

x: PCR product is not digested by either enzyme. y: PCR product is digested by the *HpaII* enzyme. z: PCR product is digested by the *MspI* enzyme. (1) refer to band presence and (0) refer to band absence. (+) refer to digestion and (−) refer to undigestion.

**Table 2:** Sequences of primers used in RAPD-PCR and the polymorphism rates in the root tissues of safflower seedlings after exposure to increasing Cu concentrations (20–1280 mg L^−1^ CuSO_4_·5H_2_O) for 21 days, compared to the control group (containing 0 mg L^−1^ Cu and irrigated only with Hoagland solution).

Primers’ Name	Polymorphism Rates (%)	Sequences (5′→3′)
OPC-01 *	16.90	TTCGAGCCAG
OPC-02 *	14.40	GTGAGGCGTC
OPC-04 *	36.40	CCGCATCTAC
*OPC-06*	52.90	GAACGGACTC
OPC-07 *	56.80	GTCCCGACGA
*OPC-08*	53.00	TGGACCGGTG
OPC-09 *	59.70	CTCACCGTCC
OPC-10 *	54.10	TGTCTGGGTG
*OPC-11*	55.90	AAAGCTGCGG
*OPA-08*	32.50	GTGACGTAGG

(*) refer to primers in which monomorphic bands were detected as a result of RAPD-PCR analyzes and used in CRED-RA analysis.

**Table 3:** Percentage changes of the genomic template stability (GTS %) found in roots of safflower seedlings after exposure to increasing copper concentrations (20–1280 mg L^−1^ CuSO_4_·5H_2_O) for 21 days.

Samples	GTS Rate (%)
20 mg L^−1^	82.75
40 mg L^−1^	88.90
80 mg L^−1^	86.26
160 mg L^−1^	83.90
320 mg L^−1^	82.25
640 mg L^−1^	84.60
1280 mg L^−1^	83.70

**Table 4:** The average rates of methylation types and patterns based on CRED-RA data analysis in the root tissues of safflower seedlings after exposure to increasing Cu concentrations (20–1280 mg L^−1^ CuSO_4_·5H_2_O) for 21 days, compared to the control group.

	Control	20 mg L^−1^	40 mg L^−1^	80 mg L^−1^	160 mg L^−1^	320 mg L^−1^	640 mg L^−1^	1280 mg L^−1^
**Type-I (%) (Non-methylation)**	2.60	3.40	4.10	**7.80** *	4.40	3.40	**2.40** *	2.60
**Type-II (%)**	3.40	3.60	3.90	5.10	5.60 *	3.30	2.60	1.40 *
**Type-III (%)**	5.10	7.20	7.40	6.60	5.10	4.30	5.30	5.90
**Type-IV (%)**	86.60 *	84.60	83.00	81.90 *	81.60 *	86.80 *	86.00	85.50
**Total methylated bands ratio (%) ^a^**	95.10	**95.40** *	94.30	93.60	**92.30** *	94.40	93.90	92.80
**Full-methylated bands ratio (%) ^b^**	91.70	**91.80** *	90.40	88.50	**86.70** *	91.10	91.30	91.40
**Semi-methylated bands ratio (%) ^c^**	3.40	3.60	3.90	5.10	**5.60** *	3.30	2.60 *	**1.40** *

^a^ Calculation of Total methylation pattern (%) = [(II + III + IV)/(I + II + III + IV)] × 100. ^b^ Calculation of Full methylation pattern (%) = [(III + IV)/(I + II + III + IV)] × 100. ^c^ Calculation of Semi methylation pattern (%) = [(II)/(I + II + III + IV)] × 100. ‘*’ is represent significance. Underlined and bold are represent max. and min. value control group; containing 0 mg L^−1^ Cu and irrigated only with Hoagland solution.

## Data Availability

Not applicable.
